# Commensal Bacteria-Specific CD4^+^ T Cell Responses in Health and Disease

**DOI:** 10.3389/fimmu.2018.02667

**Published:** 2018-11-20

**Authors:** Chiara Sorini, Rebeca F. Cardoso, Nicola Gagliani, Eduardo J. Villablanca

**Affiliations:** ^1^Immunology and Allergy Unit, Department of Medicine Solna, Karolinska Institute, Stockholm, Sweden; ^2^Department of General, Visceral and Thoracic Surgery, University Medical Center Hamburg-Eppendorf, Hamburg, Germany; ^3^Department of Medicine, University Medical Center Hamburg-Eppendorf, Hamburg, Germany

**Keywords:** commensals, microbiota, CD4^+^ T cells, inflammatory bowel disease (IBD), bacteria–host interaction, immune education, intestinal immunity, autoimmunity

## Abstract

Over the course of evolution, mammalian body surfaces have adapted their complex immune system to allow a harmless coexistence with the commensal microbiota. The adaptive immune response, in particular CD4^+^ T cell-mediated, is crucial to maintain intestinal immune homeostasis by discriminating between harmless (e.g., dietary compounds and intestinal microbes) and harmful stimuli (e.g., pathogens). To tolerate food molecules and microbial components, CD4^+^ T cells establish a finely tuned crosstalk with the environment whereas breakdown of these mechanisms might lead to chronic disease associated with mucosal barriers and beyond. How commensal-specific immune responses are regulated and how these molecular and cellular mechanisms can be manipulated to treat chronic disorders is yet poorly understood. In this review, we discuss current knowledge of the regulation of commensal bacteria-specific CD4^+^ T cells. We place particular focus on the key role of commensal-specific CD4^+^ T cells in maintaining tolerance while efficiently eradicating local and systemic infections, with a focus on factors that trigger their aberrant activation.

## Introduction

The human body is composed of a myriad of microbes that outnumber the whole body human cells (1, 2). The highest density of commensal bacteria is located in the gastrointestinal tract, which is the largest immune organ in the body (3). The vast capabilities of the microbiota have led scientists to call for a “second human genome project” to account for the effect of microbial genes in human health (4). Commensal bacteria serve various functions, from catabolizing certain food molecules to promoting tissue repair. Among other functions, the microbiota controls enteric infections by competing for common resources as well as inducing the production of antimicrobial peptides by gut epithelial cells (5, 6). Importantly, the gut microbiota shapes both local and systemic immune responses. This is most evident in the case of chronic inflammation in the intestines, as this can lead to an unbalance of immune cells as well as dysbiosis of commensals and can result in various diseases, including local inflammatory bowel disease (IBD) and systemic diseases such as diabetes, asthma, and cancer (7). While many immune cells play a role in maintaining homeostasis with the microbiota (8), both effector and regulatory CD4^+^ T cells are highly enriched in intestinal tissues and represent a major immune cell in mediating homeostatic responses. Moreover, CD4^+^ T cells are believed to be the main drivers of pathogenicity in IBD and as such are considered a main therapeutic target (9).

Commensals have been shown to play a crucial role in driving physiological CD4^+^ T cell differentiation at barrier sites. T cells recognize immunological epitopes through engagement of their T cell receptors (TCRs) with cognate antigens presented on major histocompatibility (MHC) molecules on antigen-presenting cells (APCs) (10). Unlike T cells specific for self-antigens expressed by thymic epithelial cells, commensal-specific T cells do not undergo negative selection in the thymus and are present in healthy individuals despite the constant presence of their cognate antigens (11). Importantly, T cell transfer studies have shown the ability of these cells to drive pathogenesis. In this review, we will therefore discuss the multifaceted role of commensal bacteria-specific CD4^+^ T cells with special emphasis on the regulation and fate of these cells. More in detail, we will illustrate the mechanisms at the basis of commensal-host mutualism and how their disturbance affects local and systemic health. A detailed description of the state-of-the-art models available for the study of microbiota-specific T cell responses will provide major insights into the variety of CD4^+^ T cell responses that are elicited by selected commensal bacterial species.

### Commensal-host mutualism

The relationship between humans and microbes is multifaceted; while several bacterial species can result in pathogenesis upon colonization, the vast majority are innocuous and even beneficial to the host (12). Mutualism between hosts and microbes benefits both parties but relies on a delicate balance between the antimicrobial and tolerogenic effector functions of the immune system as well as the control of pathogenicity of commensals. Commensals aid their hosts by inactivating toxins and catabolizing host-indigestible nutrients, such as some complex carbohydrates, to useful metabolites such as short chain fatty acids (SCFAs) (13–15). The commensal bacteria also control pathogen overgrowth through competition for the same biological niches and release of molecular mediators (16–22). Beyond nutrient breakdown and protection from infections, commensal bacteria also play a role in the development of anatomical structures in the intestines, angiogenesis, maturation of the epithelial layer, and leukocyte imprinting (5, 23, 24).

The gut microbiota has a vast effect on the local immune system. Accordingly, antibiotic-mediated clearance of commensal microbes has been associated with susceptibility to infections, likely due to an unfit immune system (25). Importantly, commensal bacteria can induce both pro- and anti-inflammatory CD4^+^ T cell responses via different mechanisms. For example, segmented filamentous bacteria (SFB) efficiently induce ileal IL-17 producer T helper (T_H_17) cell expansion, whereas the production of SCFAs by colonic bacteria promotes the differentiation of colon-resident regulatory T cells (26). The delicate balance between pro-inflammatory and regulatory T helper cells is believed to play a pivotal role for the maintenance of the symbiotic relationship between commensal bacteria and their host.

### Regulation of bacteria-host interaction by the mucosal barrier

The maintenance of a symbiotic relationship between commensal microbes and their host relies both on physical segregation of the microbiota to the intestinal lumen and on active sampling of bacteria by the immune system under steady-state conditions. This physical segregation is achieved through a single layer of columnar intestinal epithelial cells (IECs) kept together by tight junctions, that regulate paracellular permeability. In addition, the epithelium is covered by mucous layers of varying thickness and structure along the intestinal tract (27, 28). Disturbance of the inner colonic mucus layer structure, which can be achieved by genetic depletion of Muc2 or a short-term treatment with dextran sulfate sodium (DSS), is sufficient to allow bacteria to relocate to the epithelial layer and cause gut inflammation in mice (29–31). In CA-MLCK mice, which are characterized by constitutive tight junction barrier loss due to transgenic expression of constitutively active myosin light chain kinase, expansion of intestinal CD4^+^ T cells depends on the presence of commensal bacteria. This response resulted in protection against early *Salmonella Typhimurium* infection but exacerbated inflammation during chronic infection (32). Similarly, defective mucin production and aberrant expression of epithelial junctional proteins associated with early colorectal neoplastic lesions promoted permeability to commensal bacteria in humans, furthering inflammation and tumorigenesis (33).

**Figure 1:**
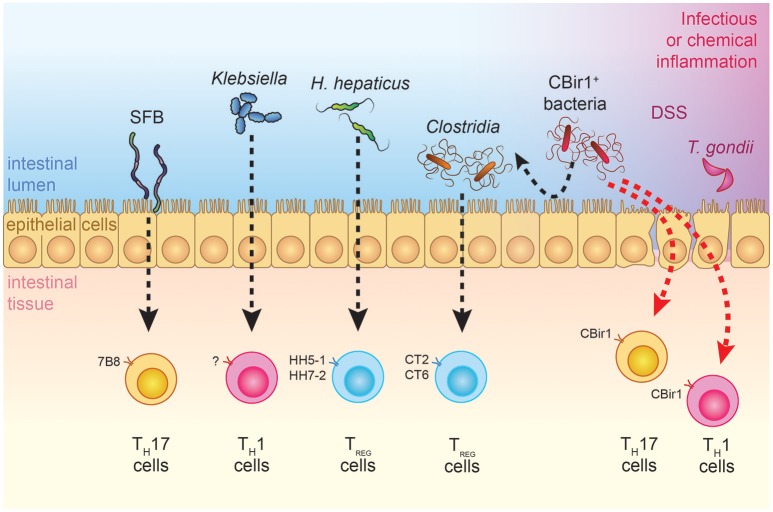
Effects of commensal bacteria on T cell differentiation during steady-state and inflammatory conditions. TCR transgenic models that are available for the study of bacteria-specific immune responses are depicted. SFB-specific 7B8 cells are preferentially skewed toward a T_H_17 cell phenotype, while *H. hepaticus*-specific TCR transgenic cells and *Clostridia*-specific CT2-CT6 cells acquire a T_reg_ fate under steady-state conditions (39, 42, 47). Physiological encounter of CBir1 T cells with their cognate antigen does not occur in adult individuals but can be triggered by different infectious or barrier-disrupting events, which shape the type of CD4^+^ T cell response (48, 49, 54). Ectopic colonization of oral *Klebsiella* in the intestines has T_H_1-inducing and pro-inflammatory effects on the gut, although antigen specificity has yet to be investigated (55).

The mucosal barrier is far from being a passive defense mechanism against microbial translocation. Immunoglobulins A (IgA), the most abundant immunoglobulin class in the body, are produced by B cells and plasma cells that reside in the Peyer's patches and intestinal lamina propria, respectively. Functional importance of this molecule in limiting commensal-specific T cell activation has been demonstrated in studies using the CBir1 TCR transgenic mouse model (Table 1). Activation of adoptively-transferred CBir1 Tg cells in response to orally-administered CBir1 flagellin was specifically blocked in WT mice, while selective impairment of IgA production or mucosal secretion unleashed CBir1 antigen-dependent T cell proliferation (48). Interestingly, IgA-mediated compartmentalization of the mucosal T cell response to the commensal microbiota does not apply to all bacteria, as activation of SFB or *Helicobacter hepaticus*-specific T cell clones occurs in immunocompetent mice (39, 42) (Figure 1). Furthermore, by the secretion of microbial peptides, epithelial cells actively contribute to the segregation of selected commensal bacteria to the intestinal lumen. These peptides are critical regulators of bacterial immunity and their impaired production is associated with intestinal and systemic inflammation. Mutations in Nod2, which are highly correlated with Crohn's disease, were found to negatively affect the production of a subgroup of intestinal anti-microbial peptides known as cryptidins (56). RegIII lectins are produced by Paneth cells in response to MyD88-dependent recognition of gut microbial patterns (57). Their depletion in RegIIIγ^−/−^ and RegIIIβ^−/−^ mice resulted in increased colonization of the intestinal epithelial surface and bacterial translocation to the liver, respectively, with consequent extra-intestinal inflammation (58, 59). Interestingly, depletion of retinoic acid (RA) receptor alpha specifically in epithelial cells resulted in increased numbers of goblet and Paneth cells and increased RegIIIγ production. In agreement with the role of RegIIIγ in regulating bacterial colonization at the epithelium vicinity, these mice did not have any detectable bacteria as seen by 16S FISH staining (60). Altogether, these data demonstrate that tight regulation of the mucosal barrier is crucial in maintaining microbial homeostasis.

**Table 1:** Models to study commensal-specific T cell responses.

**Model**	**Description**	**References**
Gnotobiotic mice	• Germ-free (GF) mice, which lack commensal microbes, have immunological defects that extend beyond the intestinal mucosa, with hypoplastic lymphoid tissues lacking B and T-cell compartmentalization	(23, 34)
	• GF mice colonized with defined bacterial species are useful to study the effects of selected microbes on the development of the immune system	(35)
	• GF mice colonized at birth with rat or human microbiota maintain an immature gut immune system compared to specific pathogen-free (SPF) mice. The immune-educating effect of the microbiota is host-specific	(36)
Tetramers	• Soluble MHC/peptide multimers with the capability of binding selected TCRs, useful for the *ex vivo* study of low-frequency endogenous antigen-specific CD4^+^ or CD8^+^ T cell populations	(37, 38)
	• I-Ab/3340-A6 tetramer allows recognition of segmented filamentous bacteria (SFB)-specific T cells	(39, 40)
	• I-Ab-CBir1p tetramer selectively stains cells that recognize CBir1 flagellin, an immunodominant microbiota antigen	(41)
	• HH1713172–86 and HH1713230–44 tetramers stain *Helicobacter*-specific T cells	(42)
TCR transgenic mice	• SFB, a unique member of Clostridiales that inhabits the terminal ileum, has been shown to specifically induce T_H_17 cell differentiation *in vivo* • In **7B8 TCR transgenic mice**, CD4 T cells recognize SFB-specific antigens • Upon transfer of naïve 7B8 cells to SFB-colonized congenic mice, these cells proliferate and are detected in the small intestinal lamina propria as T_H_17 cells	(39, 43, 44)
	• Commensal Clostridia induce the accumulation of T_reg_ cells in the intestine • **CT2 and CT6 mouse lines** express microbiota-dependent colonic T_reg_ TCRs • Upon adoptive transfer into young immunocompetent hosts, these naïve TCR Tg cells spontaneously acquired a T_reg_ cell phenotype	(45–47)
	• Increased antibodies against microbial antigens were detected in both Crohn's disease patients and animal models of gut inflammation. The Cbir1 epitope of bacterial flagellin protein was immunodominant in both settings • **CBir1 TCR transgenic mice** have CD4^+^ T cells capable of recognizing bacterial flagellin and other commensal microbes • Activation of adoptively transferred naïve Cbir1 TCR cells does not occur under steady-state conditions but can be achieved via disturbance of the intestinal mucosal barrier or lack of immunoregulatory mechanisms	(48–52)
	• **HH7-2tg and HH5-1tg mouse strains** are transgenic for clonotypic TCRs that recognize *Helicobacter hepaticus* • HH7-2tg cells acquired a Foxp3^+^RORγt^+^ regulatory phenotype under steady state conditions • Intestinal inflammation skews the *H. hepaticus*-specific response toward a pro-inflammatory T_H_17/T_H_1 cell phenotype	(42, 53)

### Regulation of CD4^+^ T cell responses against commensal bacteria

CD4^+^ T cells orchestrate the immune response through the release of pro- and anti-inflammatory cytokines and expression of co-stimulatory molecules. To this end, they play crucial roles in driving or repressing the response of macrophages, CD8^+^ T cells, and B cells toward both pathogens and autoimmune antigens [reviewed in (61)]. CD4^+^ T cells can differentiate into various T helper (T_H_) subsets with differing effector functions [reviewed in (62, 63)]. The most extensively characterized T_H_ subsets include: T_H_1 cells, which are characterized by the production of interferon gamma (IFNγ), tumor necrosis factor alpha (TNFα), and expression of the transcription factor T-box expressed in T cells (T-bet); T_H_2 cells, which produce IL-4 and IL-13 and express the transcription factor GATA-binding protein 3 (GATA-3); and T_H_17 cells, which express IL-17A/F and IL-22 and the transcription factor RA receptor-related orphan nuclear receptor RORγt. Anti-inflammatory T cell subsets include “natural” CD4^+^CD25^+^FoxP3^+^ regulatory (T_reg_) cells that develop in the thymus as well as “inducible” regulatory cells, such as FoxP3^+^ T_reg_ and FoxP3^−^ T_R_1 cells, which arise in the periphery (64–66). In addition, Bcl6-expressing T follicular helper (T_FH_) cells reside in germinal centers and coordinate B cells responses through regulation of B cell recruitment, expansion, survival, antibody class-switching, and somatic hypermutation [reviewed in (67)]. Differentiation of T cells into certain T_H_ subsets can be fostered by specific features of the microenvironment. *In vitro* studies have shown that neutralization of IFNγ reduces the development of T_H_1 cells, while transforming growth factor beta (TGFβ) promotes the differentiation of T_H_17 and T_reg_ cells (61, 68).

Adherence of selective microbes to the gut epithelium or intestinal damage can expose commensal bacterial antigens to APCs, which can then initiate commensal-specific T cell responses. Several subsets of APCs inhabit the intestinal lamina propria and have been shown to respond to fluctuations of the commensal microbiota composition (69, 70). For instance, CX3CR1^hi^ mononuclear phagocytes residing in the small intestine were reported to express tight junction proteins that allow them to extend dendrites through the intact intestinal epithelium and sample microbial antigens (71, 72). Moreover, despite being non-migratory under steady state, these APCs were able to migrate to the MLNs and trigger *Salmonella*-specific T cell responses upon dysbiotic conditions (73). Both IRF4-dependent CD103^+^CD11b^+^ dendritic cells (DCs) and CX_3_CR1^+^ intestinal macrophages have been shown to play a role in the induction of intestinal T_H_17 cells in response to epithelium-associated SFB (74, 75). A number of reports demonstrate the ability of CD103^+^CD11b^+^ DCs to produce pro-inflammatory cytokines IL-6 and IL-23 in response to detection of microbial patterns, therefore making them good candidates for the induction of T_H_17 cells (74, 76). Cytokine production by innate immune cells may synergize with or be neutralized by TCR specificity in determining the fate of the T_H_17 cell response. For instance, joint colonization with SFB and T_H_1-inducer *Listeria monocytogenes* does not prevent SFB-specific T cells from acquiring T_H_17 features (39).

Intestinal innate lymphoid cells (ILCs) represent another innate immune population with a high degree of functional compartmentalization that can be finely shaped by the composition of the commensal microbiota (77). It has been recently demonstrated that some group 3 ILCs (ILC3s) have the capacity to present antigens through MHC class II molecules, and this feature allows them to regulate the commensal-specific T cell response (78). Loss of MHCII expression within this ILC subset resulted in the accumulation and pro-inflammatory activation of commensal microbiota-specific CBir1 Tg T cells in the MLN and colonic lamina propria. In addition, the regulatory function of ILC3 was shown to rely on MHCII- and antigen-dependent induction of CBir1 T cell apoptosis, thus uncovering a novel regulatory mechanism of the commensal-specific T cell response (40).

### T cell tolerance toward commensals

Activation of T_H_ cells in the gut-associated lymphoid tissues is subjected to a delicate control, whereby T cells specific for commensal bacteria are either ignorant of their cognate antigens (e.g., CBir1 flagellin-specific cells) or physiologically skewed toward a regulatory function (e.g., CT2 and CT6 commensal-specific T cells) (45, 49) (Figure 1). Understanding the physiological mechanisms that are in place to maintain T cell homeostasis toward commensal antigens is therefore fundamental to understand why inflammation occurs and to potentially revert pathogenic activation of commensal-specific T cells.

The peculiar microenvironment present at mucosal surfaces—largely influenced by microbial metabolic products and soluble factors either digested of produced by IECs—promotes tolerance toward foreign antigens (26, 60, 79). Peripheral tolerance prevents the development of immune-mediated inflammatory diseases (IMIDs) (11, 80) and can be achieved by different mechanisms. The high frequency of T_reg_ cells in the gut, which has been shown to depend at least in part on TCR-mediated recognition of some bacteria residing in the colonic mucosa, namely *Clostridia*, provides an important mechanism for the maintenance of tolerance to commensals (45). CD4^+^ regulatory T cells dampen inflammation through the release of cytokines such as interleukin 10 (IL-10) and TGFβ. Several non-CD4^+^ T cell subsets such as CD8^+^ T cells and double negative T regulatory cells can also promote tolerance through the expression of anti-inflammatory cytokines or through direct killing of effector cells (81, 82). All of these regulatory cell types can be induced in the context of oral tolerance, which is defined as the active establishment of local and systemic unresponsiveness to antigens acquired via the oral route (83).

The gut-draining mesenteric lymph nodes (MLNs) and CD103^+^ DCs play a critical role in the induction of antigen-specific T_reg_ cells (84, 85). One mechanism of T_reg_ cell induction involves the ability of CD103^+^ DCs to metabolize dietary vitamin A to RA (86–90). The difference in T_H_ cell subsets, and in particular percentages of T_reg_ cells, along the intestinal tract could therefore, be ascribed to a differential uptake of vitamin A and other nutrients in selected anatomical locations, as well as to the presence of different consortia of commensal bacteria (45, 46, 91). While the process of tolerance toward food antigens shares some features with tolerance to commensals, the two mechanisms are fundamentally different. Indeed, tolerance to microbes is largely limited to the gut whereas oral tolerance has systemic consequences (92).

Regulatory T cells can be implicated in the maintenance of the host-microbiota commensalism at different levels. First of all, T cells that are specific for selected bacteria, i.e., *Clostridium* species or *H. hepaticus*, acquire a T_reg_ phenotype under steady-state conditions (42, 45, 47). For instance, the preferential conversion of *H. hepaticus*-specific HH7-2tg cells into functional RORγt^+^Foxp3^+^ iT_reg_ cells depends on the intrinsic expression of the transcription factor c-MAF, which is known to promote a cellular anti-inflammatory program including production of the cytokine IL-10. Selective depletion of c-MAF in T_reg_ cells resulted in the accumulation of both polyclonal and *H. hepaticus*-specific T_H_17 cells in the large intestine (42). Additionally, T_reg_ cells participate indirectly in the restriction of CBir1 flagellin-specific T cell responses by providing help to B cells which, in turn, produce CBir1-specific IgA molecules (48). Thus, T_reg_ cells contribute to tolerance of commensals by various mechanisms.

### Commensal-specific T cells in homeostasis

While the general effect of commensals on the immune system is established, it is only of recent that focus has been directed toward the specific T cells that recognize them. Antibiotic treatment has not only been shown to affect T_reg_ cell populations as a whole, but also to alter the TCR repertoire of T_reg_ cells (93). Moreover, enhanced T_reg_ suppressive activity has been observed in the presence of their cognate antigen (93). Indeed, the existence of colonic T_reg_ cells appears to be regulated by TCR specificity and antigen availability, and *in vitro* stimulation assays of colonic T_reg_-associated TCRs with host-derived commensals show that many of the local antigens these cells react to are microbiota-derived (45–47). Despite this, the microbiota does not appear to be essential to induce T_reg_ differentiation, since germ-free (GF) mice, which are devoid of microbiota, do not lack this T cell subset. The variable ontogeny of T_reg_ cells inhabiting the intestinal mucosa, either thymic-derived or peripherally induced, might explain the controversial observations regarding the effects of the presence of commensals on T_reg_ cell frequencies [reviewed in (94)]. The effect of the microbiota on T_H_1 and T_H_17 induction is more evident, as their numbers are greatly reduced in GF animals (95, 96). In particular, it has been demonstrated that the majority of T_H_17 cells in the intestinal lumen are specific for commensals (43, 46, 97).

Mouse studies reveal that commensal-specific T cells in the gut mucosa typically develop into either T_H_17 or T_reg_ cells during steady state and these subsets collectively help maintain homeostasis in these tissues. The fate of commensal-specific T cells is dependent on the microbe they are specific to. For instance, the presence of SFB, a commensal that resides in the ileum, has been shown to drive T_H_17 differentiation, whereas T cells that are specific to some *Clostridia* species develop into colonic T_reg_ cells under homeostatic conditions (43, 45, 46, 98). A comprehensive analysis of T cells from human peripheral blood and mucosal samples recently revealed that commensal-specific T cells are similarly present in healthy individuals, mainly have a memory phenotype and are capable to express different cytokines according to their immune specificities (9). In memory phase, commensal-specific CD4^+^ T cells behave like pathogen-specific CD4^+^ T cells, persisting after clearance of infection but declining in numbers over time (49, 99). Understanding the mechanisms behind the reduction of commensal-specific T cell numbers despite the persistence of their cognate antigen could shed light on the different capacity of individuals to recover from intestinal challenges (100).

Interestingly, despite the large antigenic load of commensal bacteria present within the intestinal lumen, T cells specific for certain bacterial antigens remain naïve in mice under homeostatic conditions (48). Upon disturbance of the barrier-mediated segregation of bacteria to the intestinal lumen, however, activation of T cells toward such antigens may take place. Whether activation of CD4^+^ T cell responses toward such antigens is directly contributing to the pathogenesis of inflammation or represents a mere epiphenomenon in the broader context of barrier disruption is still an open question.

## Pathogenesis of commensal-specific T cells

Local passage of either pathogenic or normally tolerated bacteria through the mucosal barrier and their systemic translocation can cause disease. As described above, despite the fact that commensal antigens are non-self, their coexistence within the host is finely regulated via multiple immune tolerance mechanisms, spanning from an efficient epithelium and mucus-mediated segregation to the secretion of antimicrobial peptides, and specific antibodies (101–103). Contact between host epithelial cells and commensals within the intestinal lumen is not in itself problematic since some bacterial species have been described to colonize the epithelial surface and induce protective immune responses in an adherence-dependent manner (43, 104).

Mice with genetic defects in the barrier develop spontaneous intestinal inflammation (105, 106) but gut-resident macrophages have been shown to efficiently clear invading pathogens thus preventing their systemic translocation (107). Intestinal immune responses are normally limited to the intestines and associated lymphoid tissue, since DCs that pick up commensal antigens migrate from the gut mucosa to the gut-draining MLNs, but not further into the body (108). However, antibodies against skin- and gut-resident commensals have been identified in humans, suggesting that we are challenged throughout life with commensal antigens and some of these immune responses have systemic effects (109). As a result of a mucosal firewall-breaching pathogen, commensal-specific T cells are activated and expand in the gastrointestinal tract (49, 110, 111) and this may result in the persistent disruption of migratory DC trafficking and impairment of tissue-specific adaptive immunity and tolerance (112). Furthermore, an acute enteric infection model has been shown to result in the differentiation of normally quiescent commensal-specific T cells into pro-inflammatory T_H_1 cells (49).

**Figure 2:**
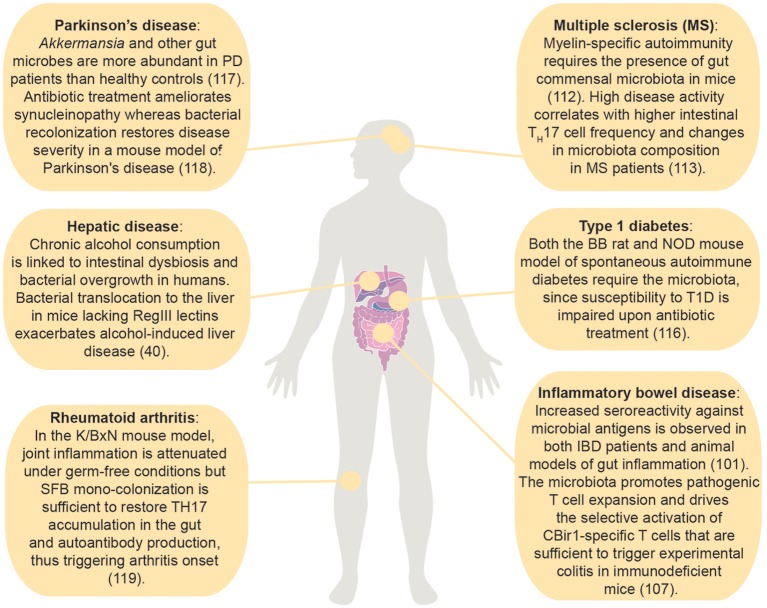
Effects of the intestinal microbiota on immune mediated diseases within and outside the gastrointestinal tract. (50); (113); (114); (115); (116); (59); (117); (51); (118).

Emerging evidence points toward the intestinal microbiota as a major factor in the establishment, persistence and/or resolution of intestinal, and extra-intestinal immune diseases (Figure 2). Although the definition of a “healthy” microbiota is still under discussion, a balanced microbiota composition is necessary to maintain intestinal immune homeostasis (36). By contrast, dysbiosis has been associated with disease by promoting immune dysregulation and inflammation in the gut and beyond. This unbalance is reflected by modifications of the immune response to physiologically-recognized bacterial antigens and novel recognition of bacteria to which the immune system is normally ignorant. For instance, pro-inflammatory T cell responses are observed toward classical T_reg_-inducer *Clostridium* species during gastrointestinal infection (49). Furthermore, immunoglobulins reactive to commensal antigens can be found in patients with active gut inflammation (50).

While the microbiota plays an important role in the determination of T cell fate, its effects are buffered by the genetic background of the host. For example, T_reg_ induction following the introduction of altered Schaedler flora (ASF) was shown to be mouse strain-dependent (119). Indeed, while IBD is recognized as a disease caused by an aberrant immune response against commensals in the intestinal mucosa, it is unknown why some people develop chronic inflammation upon barrier damage while others recover (50, 100).

### Intestinal disorders

The intestinal mucosa and its immune system, by providing the major interface between gut bacteria and the host, are susceptible to a number of different pathological conditions upon disturbance of their delicate symbiotic relationship. IBD is considered an intestinal disorder characterized by an aberrant immune response against the commensal microbiota in genetically-susceptible hosts, most likely triggered by the environment. Commensal-specific T cells are thought to play a direct role in IBD pathogenesis. For instance, transfer of naïve T cells in lymphopenic mice is sufficient to induce chronic intestinal inflammation in a microbiota-dependent manner (120, 121). Furthermore, increased seroreactivity against a commensal-derived flagellin expressed by a subset of *Clostridia* (CBir1) was found to induce a systemic adaptive immune response in both Crohn's disease patients and colitic mice, suggesting a microbiota-specific immune response (50, 122, 123). Indeed, the microbiota has been shown to promote pathogenic T cell expansion and drive the selective activation of CBir1-specific T cells that are sufficient to trigger experimental colitis in immunodeficient mice (51). Recent studies suggest that during colitis an effector T cell response is specifically activated against other microbial antigens, e.g., those provided by the mucosal-associated *Helicobacter* spp. that elicit T_reg_ responses during homeostatic conditions (53, 122). Today, several pathogenic T cells have been identified in mouse and humans, such as the IL-17 and IFNγ double-producer T cells as well as the recently described IL-22BP-producer T cells (124, 125). Whether these T cells are microbiota-specific needs to be demonstrated.

Evidence also suggests that commensal-specific T cells may play a role in the pathogenesis of colorectal cancer (CRC) and dietary conditions. Epidemiological studies reveal that chronic inflammation, such as the one resulting from IBD, increases the risk of CRC (126). The gut microbiota was shown to play a role in both stages of CRC-associated inflammation. Studies in IL-10 knockout mice showed that alteration of the microbiota by introduction of probiotic *Lactobacilli* can reduce the incidence of both mucosal inflammation and spontaneous CRC development in these mice (127). In addition, commensals have been linked to dietary conditions. For example, through their distinct metabolic patterns, some commensal bacteria are able to modify immunogenicity of dietary proteins, thus triggering gluten-specific T-cell responses and celiac disease (128). Whether bacteria-targeted therapy is effective in treating or preventing these conditions remains to be determined.

### Extra-intestinal disorders

Remarkably, gut commensal-specific T cells circulate systemically (9) and the gut microbiota affects the balance between pro-inflammatory and anti-inflammatory T cell responses also in extra-intestinal autoimmune diseases. For example, myelin-specific autoimmunity requires the presence of gut commensal microbiota, as confirmed by lack of spontaneous EAE in GF compared to SPF susceptible mice (113). Similarly, in multiple sclerosis patients, high disease activity correlated with higher intestinal T_H_17 cell frequency, and changes in microbiota composition (114). Furthermore, recent evidence unveiled the importance of the microbiota for the development of spontaneous RA (129, 130). The association between the intestinal environment and type 1 diabetes (T1D) relies on a constantly increasing number of studies both in T1D patients and mouse models of the disease. The requirement for the gut microbiota in the pathogenesis of T1D has been proven in both the BB rat and NOD mouse model of spontaneous autoimmune diabetes, whose susceptibility to the disease is impaired upon antibiotic treatment (117). Unlike in MS and RA models, however, the effect of the microbiota on the induction of pathogenic or protective immune cell responses is not unanimously accepted in T1D. However, since the antigen-specificity of microbiota-induced T cells has been poorly characterized in these disease settings, further studies are required to understand the role of commensal-specific T cells in tissue-specific autoimmune diseases. A functional link between the microbiota and Parkinson's disease (PD), a neurodegenerative disorder, was also recently delineated. *Akkermansia* and other gut microbes were more abundant in PD patients than healthy controls (115). Antibiotic treatment was capable of ameliorating synucleinopathy (motor dysfunctions derived from the aggregation of the protein α-synuclein) in a mouse model of Parkinson's disease, and recolonization of adult mice with commensal bacteria derived from PD patients or administration of selected bacterial metabolites, namely SCFAs, was sufficient to restore disease penetrance (116). Whether or not reestablishment of a normal microbiota composition can revert PD and other neurogenerative conditions remains to be determined.

## Concluding remarks

The use of experimental mouse models has provided key insights into the complex regulation of intestinal immune homeostasis by the microbiota. Descriptive studies comparing IBD patients and healthy individuals have implicated CD4^+^ T cell-mediated immune responses against commensal bacteria as a potential key factor in the progression of the disease. Moreover, emerging evidence points toward far-reaching consequences of altered microbiota-mediated T cell responses originated in the gut. For instance, aberrant T cell differentiation in the gut have shown effects in skin tumor growth, the onset of T1D as well as MS. Hence, it is broadly accepted that, besides its gut-centric effects, the microbiota has also extra-intestinal repercussions on its host. The role of commensal-specific T cells in various disease settings is yet to be determined and only with deeper understanding of the molecular and cellular mechanism driving commensal specific T cell differentiation and function will the goal of targeting T cell responses to cure IBD become feasible. Toward this goal, the advances in microbiota analysis, use of gnotobiotic mice and TCR transgenic experimental models provides the potential to discern such mechanisms. Moreover, progress in available tools to track commensal specific T cells in humans are urgently needed. Accomplishing these goals will require tight collaboration between researchers and the clinics.

## Author contributions

CS, RFC, and EJV outlined and wrote the manuscript. CS and EJV designed and made the figures. NG revised the manuscript. All authors approved the manuscript for publication.

### Conflict of interest statement

The authors declare that the research was conducted in the absence of any commercial or financial relationships that could be construed as a potential conflict of interest.
